# 12-Deoxyphorbol-13-Hexadecanoate Abrogates OVX-Induced Bone Loss in Mice and Osteoclastogenesis *via* Inhibiting ROS Level and Regulating RANKL-Mediated NFATc1 Activation

**DOI:** 10.3389/fphar.2022.899776

**Published:** 2022-06-03

**Authors:** Qi He, Junzheng Yang, Delong Chen, Yejia Li, Dawei Gong, Hui Ge, Zihao Wang, Haibin Wang, Peng Chen

**Affiliations:** ^1^ First School of Clinical Medicine, Guangzhou University of Chinese Medicine, Guangzhou, China; ^2^ The Laboratory of Orthopaedics and Traumatology of Lingnan Medical Research Center, Guangzhou University of Chinese Medicine, Guangzhou, China; ^3^ Department of Orthopaedic Surgery, Erasmus University Medical Center, Rotterdam, Netherlands; ^4^ Department of Orthopedics, Shunde Hospital, Guangzhou University of Chinese Medicine, Foshan, China; ^5^ Department of Orthopedics, Wendeng Orthopaedic and Traumatologic Hospital of Shandong Province, Weihai, China; ^6^ Department of Orthopedics, Guangzhou Hospital of Integrated Chinese and Western Medicine, Guangzhou, China; ^7^ Queen’s University Belfast, Belfast, United Kingdom; ^8^ Department of Orthopaedics, The First Affiliated Hospital, Guangzhou University of Chinese Medicine, Guangzhou, China

**Keywords:** 12-deoxyphorbol-13-hexadecanoate, MAPK signaling pathway, Bone loss, ROS, NFATc1

## Abstract

Osteoporosis is a major health problem in the elderly. Almost every bone can fracture due to the increased bone fragility in osteoporosis, posing a major challenge to public health. 12-Deoxyphorbol-13-hexadecanoate (DHD), one of the main bioactive components of *Stellera chamaejasme* L. (Lang Du), is considered to have antitumor, antibacterial, and antifungal properties. However, the role of DHD in osteoporosis is still elusive. In this study, we demonstrated for the first time that DHD inhibits the receptor activator of nuclear factor-κB ligand (RANKL)-induced osteoclastogenesis and bone resorption in a dose- and time-dependent manner without exhibiting cytotoxicity *in vitro*. Mechanistically, we found that DHD not only represses the expression of osteoclasts marker genes by suppressing RANKL-induced mitogen-activated protein kinase (MAPK) and calcium signaling pathways but also scavenges reactive oxygen species (ROS) through enhancing cytoprotective enzymes expression. Furthermore, DHD inhibits the activation of nuclear factor of activated T cells 1 (NFATc1) during RANKL-induced osteoclasts formation. Preclinical studies revealed that DHD protects against bone loss in ovariectomy (OVX) mice. In sum, our data confirmed that DHD could potentially inhibit osteoclastogenesis by abrogating RANKL-induced MAPK, calcium, and NFATc1 signaling pathways and promoting the expression of ROS scavenging enzymes, thereby preventing OVX-induced bone loss. Thus, DHD may act as a novel therapeutic agent to manage osteoporosis.

## Introduction

The skeletal system is the hardest tissue in the whole body, providing the protection and support functions with normal bone density (Buck and Dumanian, 2012). However, some bone diseases, including osteoporosis, impair bone density and bone strength *via* disturbing bone remodeling homeostasis. As a systemic bone metabolic condition, osteoporosis is characterized by a reduction of overall bone mass and bone microarchitecture impairment, leading to an increment in bone fragility and higher fracture risk (Lorentzon and Cummings, 2015). Osteoporosis is more common in the elderly population and remains the leading cause of death among older individuals worldwide (Reginster and Burlet, 2006). By the year 2050, the global population of over 65 age is predicted to reach 1.555 billion, and meanwhile, the number of fractures caused by osteoporosis will rise to 6.26 million (Shanb and Youssef, 2014). Unfortunately, there are currently very limited medications available for osteoporosis prevention and treatment. Drugs such as estrogen (Manson et al., 2003), parathyroid hormone (Tang et al., 2019), calcitonin (Sondergaard et al., 2010), and bisphosphonates (Zhang et al., 2019) are clinically utilized to treat osteoporosis. They help inhibit the differentiation and function of osteoclasts (OCs) and maintain bone mineral density, aiming to reduce bone fractures (Tella and Gallagher, 2014). These medications can lead to serious side effects containing elevated blood pressure, raised breast cancer risk, and hypercalcemia. Accumulating evidence indicates that natural compounds with fewer side effects are more safe and efficient for chronic diseases than synthetic drugs. Hence, there is an urgent need to investigate natural substances in managing osteoporosis.

Numerous studies have proven that Chinese herbal medicine (CHM) is an effective strategy for alleviating osteoporosis symptoms (Liu et al., 2019). *Euphorbia* fischeriana Steud., a perennial herbaceous plant in the *Euphorbiaceae* family (spurge family), is a traditional Chinese medicinal plant (Sun and Liu, 2011). The root of *Euphorbia* fischeriana Steud., known as *Stellera chamaejasme* L. (Thymelaeaceae) or “Rui Xiang Lang Du,” has been mainly used for the treatment of cancer, edema, abdominal distension, skin disorders, and tuberculosis (Wang et al., 2016; Jiang and Li, 2018). Up to data, several types of compounds have been isolated from *Stellera chamaejasme* L., including diterpenes, triterpenes, and phenolic acids (Jian et al., 2018). These chemical constituents exhibit various pharmacological properties, such as antitumor, anti-inflammatory, antioxidant, antibacterial, immune-enhancing, prevention, and treatment of osteoporosis (Sun and Liu, 2011; Ma et al., 2014; Wang et al., 2015). Even though previous studies have reported the role of those extractions in protecting osteoporosis, almost all of them are triterpenes and steroid compounds. 12-Deoxyphorbol-13-hexadecanoate is a diterpenoid compound isolated from *Stellera chamaejasme* L. (Thymelaeaceae) with higher biological activity (Liu et al., 1996). However, the role of DHD in bone remodeling unbalance conditions is very rare.

Bone homeostasis is regulated by balancing osteoblast-induced bone formation and osteoclasts-induced bone resorption (Alippe et al., 2017). Osteoporosis is widely acknowledged to be the result of OCs hyperactivation. OCs are multinucleated cells in bone marrow, which are derived from bone marrow monocytes (BMMs) (Adachi et al., 2011). In pathological conditions, osteoclasts precursors enter blood circulation under the action of chemokines and reach the bone surfaces where BMMs are differentiated into osteoclasts under stimulation of receptor activator of nuclear factor-κB ligand (RANKL) and macrophage colony-stimulating factor (M-CSF) (Kalbasi Anaraki et al., 2015). Compelling evidence has confirmed that M-CSF and RANKL are required for osteoclasts differentiation (Nakagawa et al., 1998; Ono and Nakashima, 2018). Excessive production of these cytokines leads to promoting osteoclasts differentiation and improves abnormal bone resorption capacity, which can result in a large amount of bone loss in osteoporosis. Furthermore, increasing the level of reactive oxygen species (ROS) in BMMs also promotes osteoclastogenesis and osteoclastic activation (Yamasaki et al., 2009; Manolagas, 2010). The binding of RANKL to receptor activator of nuclear factor-κB (RANK) induces its intramembranous portion to react rapidly with tumor necrosis factor receptor-associated factor 6 (TRAF6), leading to the activation of MAPK and Ca^2+^ signaling pathways, which are then followed by the initial induction and activation of c-Fos and nuclear factor of activated T cells 1 (NFATc1) (Lorenzo, 2017; Ikebuchi et al., 2018).

Under the pharmacological consensus (Heinrich et al., 2020), we composed this study and assumed that DHD might be involved in suppressing RANKL-mediated osteoclasts formation and its downstream molecular mechanism. Our results confirmed that DHD could potentially inhibit osteoclastogenesis by abrogating RANKL-induced MAPK, calcium, and NFATc1 signaling pathways and promoting the expression of ROS scavenging enzymes, thereby preventing OVX-induced bone loss.

## Materials and Methods

### Materials and Reagents

**FIGURE 1:**
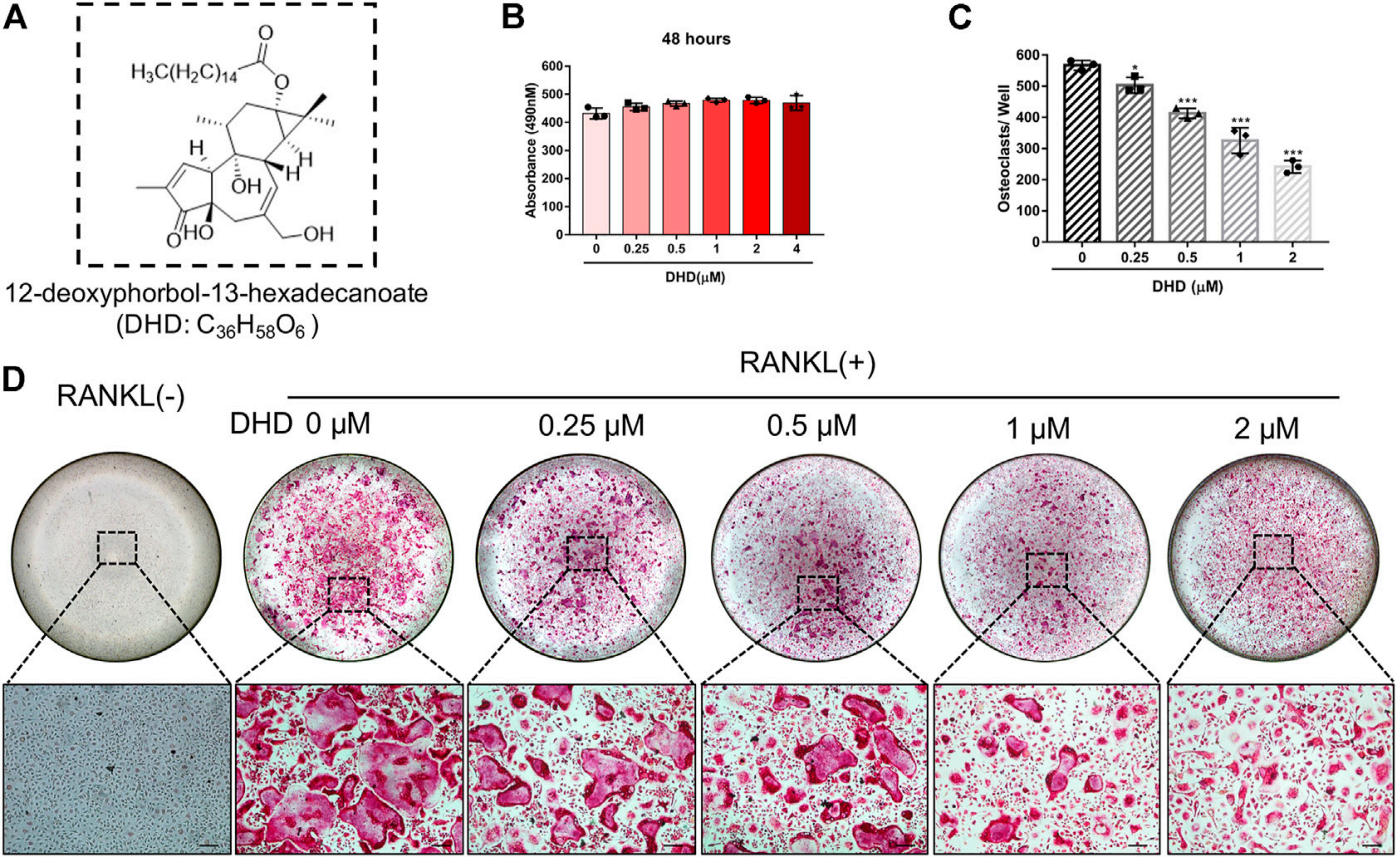
DHD abrogates RANKL-induced osteoclastogenesis in a dose-dependent manner. **(A)** Chemical structure of DHD. **(B)** The effects of the different concentrations of DHD (0, 0.25, 0.5, 1, 2, and 4 μM) on BMMs for 48 h were measured by an MTS assay. **(C)** The number of osteoclasts per well was quantitatively analyzed. Cells with TRAcP^+^ and multinucleated (>3 nuclei) were identified as osteoclasts. **(D)** Representative images of TRAcP staining after treatment with DHD at increasing concentrations (scale bar = 200 μm). Data were presented as means ± SD; ∗*p* < 0.05, and ∗∗∗*p* < 0.001 relative to RANKL-induced controls.

DHD with a purity of >95% (Figure 1A) was obtained from Sun Yat-Sen University (Guangzhou, China), and the stock solution was 100 mM DHD in dimethyl sulfoxide (DMSO, Sigma-Aldrich). The working solution was achieved with cell medium *in vitro* study and PBS *in vivo* study. Alpha modified minimum essential medium (α-MEM), rhodamine-conjugated phalloidin, penicillin/streptomycin (P/S), and fetal bovine serum (FBS) were acquired from Thermo Fisher Scientific (Scoresby, Australia). RANKL (receptor activator of nuclear factor-κB ligand) and M-CSF (macrophage colony-stimulating factor) were obtained from PeproTech Inc. (United States). DAPI solution was bought from Santa Cruz Biotechnology (San Jose, CA, United States). The 3-(4,5-Dimethylthiazol-2-yl)-5-(3-carboxymethoxyphenyl)-2-(4-sulfophenyl)-2H-tetrazolium salt (MTS) solution was obtained from BestBio (Shanghai, China).

Promega provided the luciferase analysis reagents (Sydney, Australia). Tsingke Biological Technology supplied the oligo-dT primer (Beijing, China). Accurate Biology provided the SYBR green master mix and the Evo M-MLV RT Kit (Guangzhou, China). Primary antibodies were brought from Santa Cruz Biotechnology (San Jose, CA, United States) for NFATc1 (sc-7294), *β*-actin (sc-47778), Cathepsin K (sc-48353), V-ATPase-d2 (sc-517031), and IκB-α (sc-371). Cell Signaling Technology provided primary antibodies against c-Fos (Cat# 2250S), HO-1 (Cat# 70081S), p38 (Cat# 9212L), p-JNK1/2 (Cat# 9252S), p-P38 (Cat# 4511L), catalase (Cat# 12980S), and JNK1/2 (Cat# 9252L) (Beverly, MA, United States). Sigma Aldrich provided the TRAcP staining kit (Sydney, Australia). Reactive Oxygen Species Assay Kit (S0033S) was purchased by Beyotime Biotechnology Company (Guangzhou, China).

### Cell Culture

Primary bone marrow monocytes (BMMs) were isolated from the femoral and tibial bone marrows of six-week-old C57BL/6 J mice that were euthanized through cervical dislocation. The isolated cells were passed to a fresh 75-cm^2^ culture flask containing complete α-MEM (plus 10% FBS, 1% penicillin/streptomycin, and 50 ng/ml M-CSF) and incubated at 37°C in a 5% CO_2_ atmosphere. BMMs from passages two to five were used in the experiments, and the medium was refreshed every 2 days. RAW264.7 cells (mouse macrophage cells) were bought from the American Type Culture Collection (ATCC, Manassas, VA) and cultured in complete α-MEM containing 10% FBS, 100 U/ml of penicillin, and 100 μg/ml of streptomycin. The growth medium was replaced every other day. Cells were subcultured when they were 80%–90% confluent.

### TRAcP Staining

To induce osteoclasts differentiation, BMMs were plated overnight at a density of 5 × 10^3^ cells/well on a 96-well plate with M-CSF (50 ng/ml). The following day, BMMs were categorized into a control group and treated groups with RANKL (50 ng/ml) and incremental concentrations of DHD (0, 0.25, 0.5, 1, and 2 μM) for 5 days. Then, the cells were stained with TRAcP after being treated with a 2.5% glutaraldehyde solution. Multinucleated (TRAcP-positive) cells with more than three nuclei were recognized as osteoclasts.

### Cytotoxicity Assay

The MTS assay was introduced to detect the effect of DHD on cell viability. BMMs were plated into a 96-well plate at a density of 5 × 10^3^ cells/well and then incubated in complete α-MEM with 50 ng/ml M-CSF and with diverse concentrations of DHD for another 48 h. Thereafter, the 96-well plate was incubated in a 37°C incubator for 2 h upon the addition of 10 μL of MTS solution in each well. The absorbance was measured using the BMG plate reader (Ortenberg, Germany) at 490 nm of wavelength.

### Immunofluorescence Staining of Vinculin and F-Actin Belts

BMMs were cultured overnight at a density of 5 × 10^3^ cells/well in 96-well plates with complete α-MEM and 50 ng/ml M-CSF. Subsequently, BMMs stimulation was done using 50 ng/ml RANKL for mature osteoclasts formation in the presence or absence of various DHD concentrations (1 and 2 μM). Then, cells were fixed in 4% paraformaldehyde (PFA) for 10 min and permeabilized for 5 min with 0.1% Triton X-100. After that, cells were blocked in phosphate-buffered saline (PBS) containing 3% bovine serum albumin (BSA) for half an hour and then underwent incubation in the darkroom for 45 min for F-actin ring staining with rhodamine-conjugated phalloidin. BMMs were incubated overnight at 4°C with Vinculin Recombinant mouse monoclonal primary antibody (Invitrogen, Rockford, Illinois, United States) and then labeled at room temperature for 45 min with Alexa Fluor 488-conjugated goat anti-mouse IgG secondary antibody (Invitrogen, Rockford, Illinois, United States). After being rinsed with PBS and counterstained with DAPI (Santa Cruz, United States), images were captured using a confocal fluorescence microscope (Nikon, A1 PLUS, Tokyo, Japan).

### Luciferase Reporter Assay

RAW264.7 cells were stably transfected with two kinds of luciferase reporter constructs [NFATc1 and Nrf2-antioxidant response element (ARE)], which respond to NFATc1 and ARE, respectively (van der Kraan et al., 2013; Hong et al., 2020). Equal numbers of 1.5 × 10^5^ transfected RAW264.7 cells were cultured in each well of a 48-well plate to anchor overnight and then were pretreated with varying different DHD concentrations (0, 0.25, 0.5, 1, and 2 μM) for 60 min. Thereafter, cells were stimulated with 50 ng/ml RANKL for 24 h (for analysis of NFATc1) or 48 h (for detection of ARE). Then, the cell lysis was prepared for luciferase activity evaluation *via* the Promega luciferase kit (Promega, Sydney, NSW, Australia) and BMG Polar Star Optima luminescence reader (BMG; Labtech, Offenburg, Germany).

### Hydroxyapatite Uptake Assay

In order to assess the resorptive ability of osteoclasts, the hydroxyapatite resorption assay was introduced as described in our previous studies (Chen et al., 2020; Chen et al., 2021). For this, 1 × 10^5^ BMMs cells were equally distributed in the six-well collagen-coated plates (BD Biosciences) and induced by 50 ng/ml M-CSF and RANKL to form mature osteoclasts for 3 days. Afterward, we detached the cells gently and plated them into individual wells on a 96-well hydroxyapatite-coated plate (Corning Osteoassay, Corning, NY). The mature osteoclasts were then grown in complete α-MEM without or with DHD (1 and 2 μM) in the presence of M-CSF and RANKL. After 48 h, half well cells were stained by TRAcP solution to calculate the OCs numbers. The remaining wells were bred with 10% bleach solution 10 min to be unanchored, facilitating the resorbed hydroxyapatite regions measurement through a Nikon standard light microscope (Nikon Corporation). The Image J software was fully utilized to quantify the hydroxyapatite surface areas resorbed by osteoclasts.

### Intracellular Ca^2+^ Oscillation Measurement

The Ca^2+^ sensitive fluorescent dye Fluo4-AM (Molecular probes, Thermo Fisher Scientific, Scoresby, Australia) was employed to quantify intracellular Ca^2+^ oscillations relying on the manufacturer’s protocol. Briefly, BMMs were cultured at a density of 1.5 × 10^4^ cells in a 48-well plate with M-CSF (50 ng/ml). The following day, the cells were exposed to 2 μM DHD for 1 h and then stimulated for 24 h by RANKL (50 ng/ml). After rinsing in calcium assay buffer (250 mM probenecid and 1% FBS in HANKS balanced salt solution), the cells were bred with 4 μM Fluo4 solution (Fluo4-AM dissolved in 20% Pluronic-F127 (w/v) and DMSO as a stock solution, light-protected) in a 37°C warm box for 45 min. The cells were left at room temperature for 20 min following another rinse with calcium assay buffer. The fluorescence intensity was investigated at 488 nm wavelength through an inverted fluorescent microscope (Nikon, Tokyo, Japan). Image acquisition was accomplished by a microscope after every 2 s until 3 min. Cells with two or more recorded peaks were counted to be oscillating cells. The oscillation intensity was quantified by deducting the lowest intensity from the top intensity in each group.

### Total RNA Isolation and RT-qPCR Analysis

Quantitative reverse transcription PCR (RT-qPCR) was used to quantify the mRNA expression of osteoclasts-specific and ROS-related genes. The total RNA of BMMs treated without or with different concentrations of DHD (1 and 2 μM) in the presence of RANKL (50 ng/ml) was isolated in six-well plates using Evo M-MLV RT Kit. Nanodrop 2000 was utilized for total RNA quality measurement. cDNA was reverse-transcribed from 500 ng total RNA. Real-time PCR was performed using SYBR Green Pro Tap usage in a ViiA™ 7 real-time PCR equipment (Applied Biosystems, United Kingdom). The parameters of RT-qPCR were 30 cycles of 40 s at 94°C, 60 s at 60°C, and 40 s at 72°C, as well as a final extension step of 72°C 5 min (He et al., 2021). The 2^−ΔΔCt^ technique was utilized to compute the relative mRNA expression levels of target genes through the utilization of 18 s as an internal control. The specific primers used are listed in Table 1.

**TABLE 1:** Quantitative real-time PCR primer sequences.

Genes	Forward (5′-3′)	Reverse (5′-3′)
*Acp5*	GCG​ACC​ATT​GTT​AGC​CAC​ATA​CG	CGT​TGA​TGT​CGC​ACA​GAG​GGA​T
*Nfatc1*	GGT​GCC​TTT​TGC​GAG​CAG​TAT​C	CGT​ATG​GAC​CAG​AAT​GTG​ACG​G
*c-Fos*	GGG​AAT​GGT​GAA​GAC​CGT​GTC​A	GCA​GCC​ATC​TTA​TTC​CGT​TCC​C
*Ctr*	AAG​ATG​GAC​CCT​CAT​GCC​AGT​G	CTC​GTC​GGT​AAA​CAC​AGC​CAT​G
*Nrf2*	CAG​CAT​AGA​GCA​GGA​CAT​GGA​G	GAA​CAG​CGG​TAG​TAT​CAG​CCA​G
*Keap1*	ATC​CAG​AGA​GGA​ATG​AGT​GGC​G	TCA​ACT​GGT​CCT​GCC​CAT​CGT​A
*Ho-1*	CAC​TCT​GGA​GAT​GAC​ACC​TGA​G	GTG​TTC​CTC​TGT​CAG​CAT​CAC​C
*Catalase*	CTC​GCA​GAG​ACC​TGA​TGT​CC	GAC​CCC​GCG​GTC​ATG​ATA​TT
*18s*	TGG​TTG​CAA​AGC​TGA​AAC​TTA​AAG	AGT​CAA​ATT​AAG​CCG​CAG​GC

### Western Blotting

To analyze the osteoclasts-related proteins at early time points, BMMs were seeded at a density of 2 × 10^5^ cells/well with serum starvation for 2 h in six-well plates and then pretreated with DHD (2 μM) for 1 h prior to RANKL induction for the stated time points (0, 5, 10, 20, 30, and 60 min). In long-time course protein expression detection, BMMs were incubated in a six-well plate at a concentration of 1 × 10^5^ cells/well and were stimulated by 50 ng/ml RANKL and M-CSF in the presence or absence of DHD (2 μM) for 0, 1, 3, and 5 days. For assessing the expression of ROS-related proteins, BMMs (1.5 × 10^5^ cells/well) were cultured in six-well plates with or without different concentrations of DHD (0, 1, and 2 μM) for 2 days. Cell proteins were extracted and quantified *via* the radioimmune precipitation assay (RIPA) lysis buffer and BCA protein assay kit (Beyotime). Then, the proteins were loaded in 10% sodium dodecyl sulfate-polyacrylamide gel electrophoresis (SDS-PAGE) and transferred to nitrocellulose membranes (GE Healthcare, Silverwater, Australia). After a three-time wash with Tris-buffered saline Tween 20 (TBST) buffer (Sigma, America), the membranes were blocked with 5% skimmed milk for 1.5 h at room temperature. The following primary antibodies: anti-NFATc1, anti-c-Fos, anti-V-ATPase-d2, anti-CTSK, anti-pJNK1/2, anti-JNK1/2, anti-pP38, anti-P38, anti-catalase, anti-HO-1, and anti-β-actin (1:1000) were incubated with membranes in the cold room. The following day, the membranes were rinsed with TBST three times for 5 min each and underwent incubation at room temperature for 2 h with horseradish peroxidase-conjugated secondary antibodies (1:3000). In order to visualize the immunological response, the membranes were exposed to chemiluminescence HRP substrate (Millipore, Merck, United States). The images were taken with the aid of a Bio-Rad gel imaging system.

### Detection of Intracellular ROS

Reactive Oxygen Species Assay Kit was used to detect the intracellular ROS activity. BMMs were cultured in a six-well plate at a density of 2 × 10^5^ cells/well overnight. The following day, the medium was replaced with 50 ng/ml RANKL and DHD at different concentrations (0, 1, and 2 μM). After 48 h, BMMs were harvested and washed with serum-free medium three times and then stained with dichloro-dihydro-fluorescein diacetate in a dark room for 20 min. The excitation wavelength of 488 nm and an emission wavelength of 525 nm were analyzed *via* FACS LSRFortessaTM flow cytometer (BD Biosciences, Franklin Lakes, NJ).

### Mouse Ovariectomy Experiments

The 8-week-old female C57BL/6J mice (*n* = 15, 20.21 ± 0.10 g) were provided by the Animal Laboratory Center of Guangzhou University of Chinese Medicine [SCXK (Yue) 2018–0034]. All mice were randomly allocated to three groups: sham (*n* = 5), ovariectomy (OVX) (*n* = 5), and OVX + DHD (2 mg/kg DHD, *n* = 5) groups. The mice were retained on an alternating 12 h light/dark cycle in a specific-pathogen-free (SPF) environment with a relative humidity of 55%–60% and temperature of 22°C–25°C (Laboratory Animal Center, The First Affiliated Hospital of Guangzhou University of Chinese Medicine, SYXK (Yue) 2018–0092). After 1 week of acclimation, the mice in the OVX and OVX + DHD groups underwent bilateral oophorectomy procedure, whereas the sham models were archived by resecting similar-sized adipose tissue around the ovaries region (Nakagawa et al., 1998). Seven days after surgery, intraperitoneal injections of DHD (2 mg/kg) were administered every 2 days to the OVX + DHD group and PBS to the sham and OVX groups for 6 weeks (Ono and Nakashima, 2018). The mice were then sacrificed through cervical dislocation 1 day after the final injection, and the femurs were collected for further experiments. The Institutional Animal Ethics Committee of The First Affiliated Hospital of Guangzhou University of Chinese Medicine accepted all *in vivo* experimental procedures utilized in this study (Ethic NO. TCMF1–20201202001).

### Micro-CT Scanning

The mice’s left femurs were harvested and fixed for 24 h in 4% paraformaldehyde. Micro-CT equipment (Skyscan, Bruker, Belgium) was exploited to scan the femurs using the following parameters (Chen et al., 2020): 0.4-degree rotation step, 0.5 mm thick aluminum filter, 50 kV voltage, 9 μm pixel size, and 500 μA source current. A volume of interest (VOI, 1 mm) at 1.5 mm on top of the femoral growth plate for analyzing femur trabecular is chosen. Also, the cortical bone analysis was performed on the top 5 mm VOI (1 mm). The structural variables were utilized to assess the trabecular bone, including bone volume/tissue volume (BV/TV, %), trabecular number (Tb.N, 1/mm), trabecular spacing (Tb.Sp, mm), and trabecular thickness (Tb.Th, mm). Cortical bone parameters were observed by measuring cortical bone area (Ct.Ar, mm^2^), total tissue area (Tt.Ar, mm^2^), cortical area fraction (Ct.Ar/Tt.Ar, %), and cortical thickness (Ct.Th, mm) (Krause et al., 2020). The CT Analyzer software evaluated the above data (Bruker micro-CT, Kontich, Belgium).

### Statistical Analysis

All experiments were carried out at least three times independently. The data in our study were expressed as mean ± standard deviation (SD). The Student’s *t*-test and ANOVA were utilized for statistical analysis. The variations were deemed statistically significant (*p* < 0.05).

## Results

### DHD Attenuates RANKL-Induced Osteoclastogenesis *In Vitro*

**FIGURE 2:**
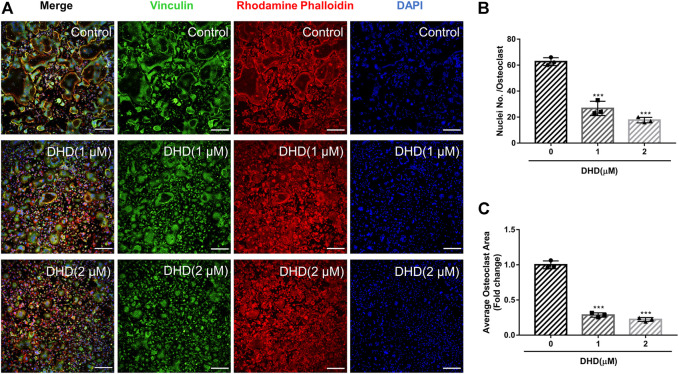
DHD represses RANKL-induced osteoclasts fusion. **(A)** Representative confocal images of osteoclasts stained for vinculin (green), F-actin belts (red) and nuclei (blue) (scale bar = 200 μm). **(B,C)** Quantification of the osteoclasts per area and the mean number of nuclei per in each cell.

**FIGURE 3:**
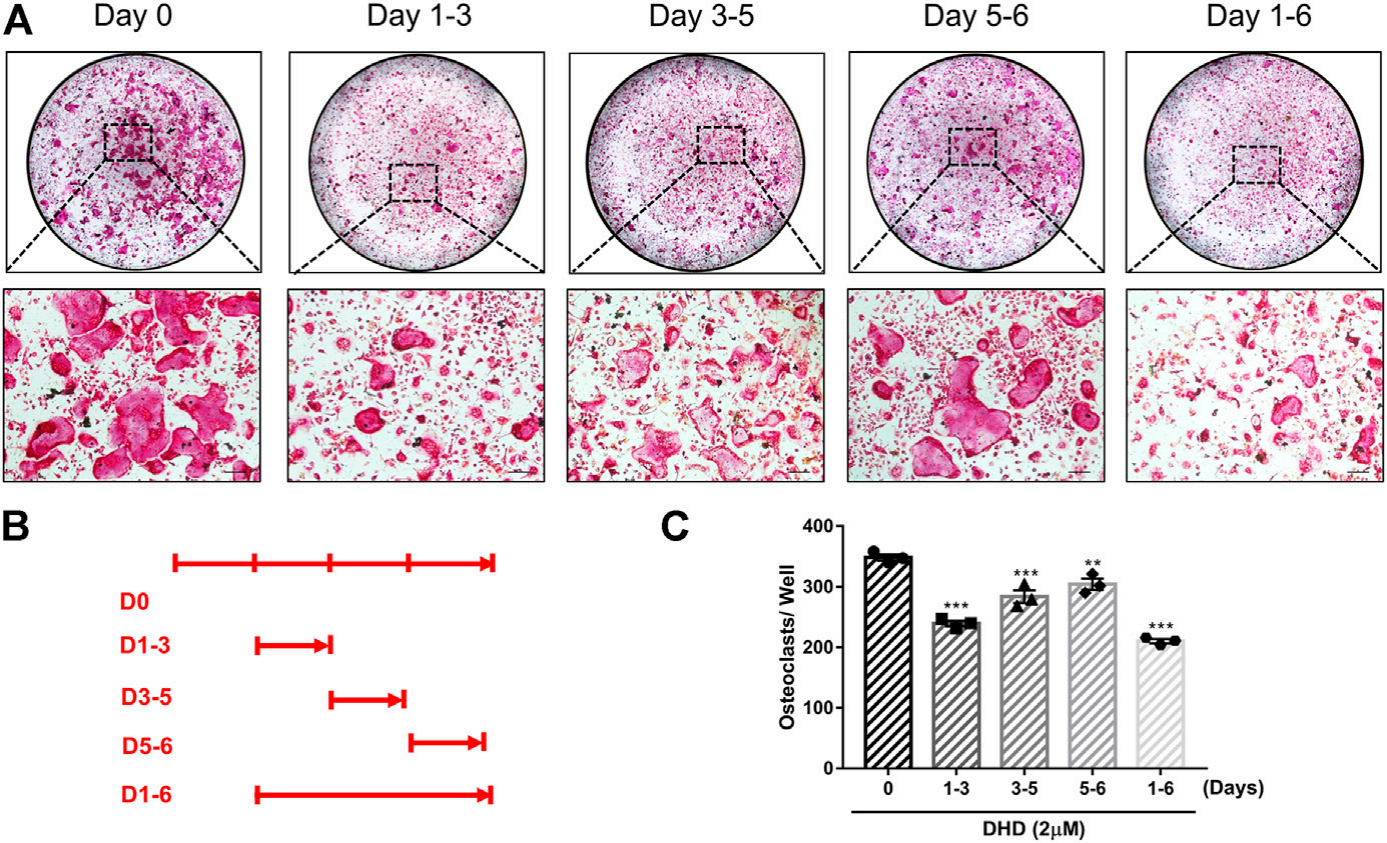
DHD suppresses RANKL-induced osteoclastogenesis in the early, middle and later stages. **(A)** Representative images of TRAcP^+^ cells treated with 2 μM DHD at different time points (scale bar = 200 μm). **(B)** Time points of DHD treatment on BMMs. **(C)** Multinucleated cells (>3 nuclei) treated with DHD at the indicated days were quantitatively analyzed for osteoclasts formation.

Cell viability was evaluated by conducting the MTS assay. No cytotoxic effects of DHD on osteoclasts were found at the concentration range in our study to rule out whether cell cytotoxicity of DHD might be implicated in suppressing osteoclasts formation (Figure 1B). To assess the DHD potential effect in RANKL-induced osteoclastogenesis, freshly isolated BMMs were stimulated to form osteoclasts in the absence or presence of DHD at various concentrations. The results showed that, in a dose-dependent manner, TRAcP-positive multinucleated osteoclasts formation was significantly inhibited by DHD at the concentration of 1 and 2 μM (Figures 1C,D). Fibrous actin (F-actin) rings are typical cytoskeletal structures of mature OCs. To verify the effect of DHD on the fusion of osteoclasts precursors, we investigated morphological changes in F-actin cytoskeletal and vinculin structures, as well as the nuclei numbers per osteoclasts and the osteoclasts average areas (Figures 2A–C). Following DHD intervention, F-actin rings and vinculin belts in osteoclasts became smaller, and the nuclei numbers declined in contrast to the control group (Figure 2A). Moreover, following the DHD treatment at 1 and 2 μM, the average osteoclasts area and the numbers of nuclei for each osteoclasts were significantly decreased (Figures 2B,C). To further determine the most sensitive stage of osteoclasts formation responding to DHD treatment, 2 μM DHD was utilized at different osteoclastogenesis stages (Figure 3). As shown in Figure 3C, DHD treatment at all three stages resulted in interrupting the formation of osteoclasts and was remarkably significant in the early stages (days 1–3) of osteoclasts differentiation. Overall, the above data indicated that DHD has no obvious cytotoxicity to the osteoclasts precursors and mainly affects the early stage of RANKL-induced osteoclasts differentiation.

### DHD Represses Osteoclasts Resorptive Activity and Expression of Osteoclasts-Specific Genes

**FIGURE 4:**
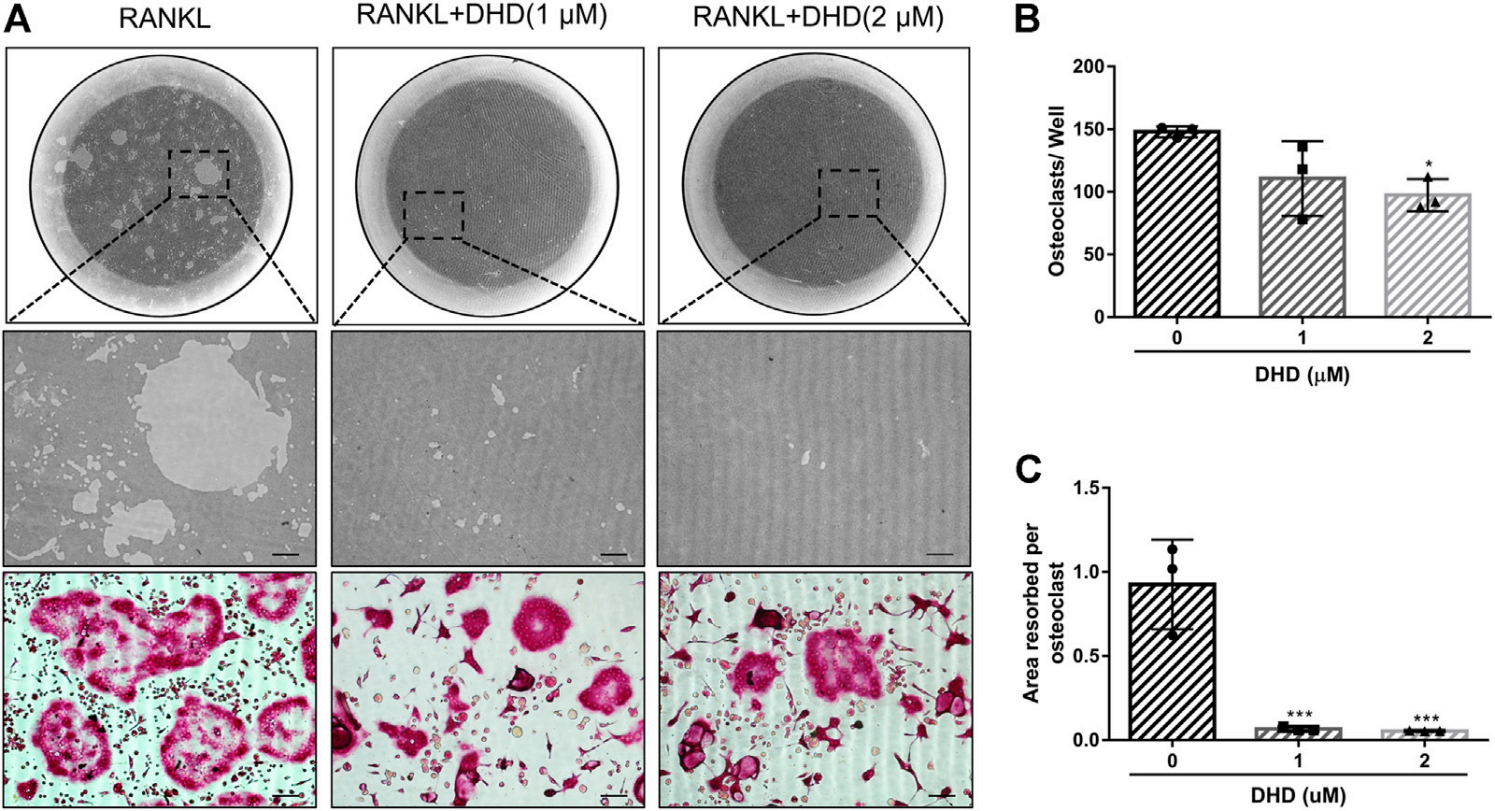
DHD suppresses osteoclastic bone resorption activity. **(A)** Representative images of eroded regions and TRAcP-stained cells on hydroxyapatite-coated plates in the presence or absence of 1 and 2 μM DHD (scale bar = 200 μm). **(B)** Quantitative analysis of osteoclasts in each well (96-well plate). **(C)** Quantitative analysis of resorbed proportion per osteoclasts after treatment with the indicated concentrations of DHD (1 and 2 μM).

**FIGURE 5:**
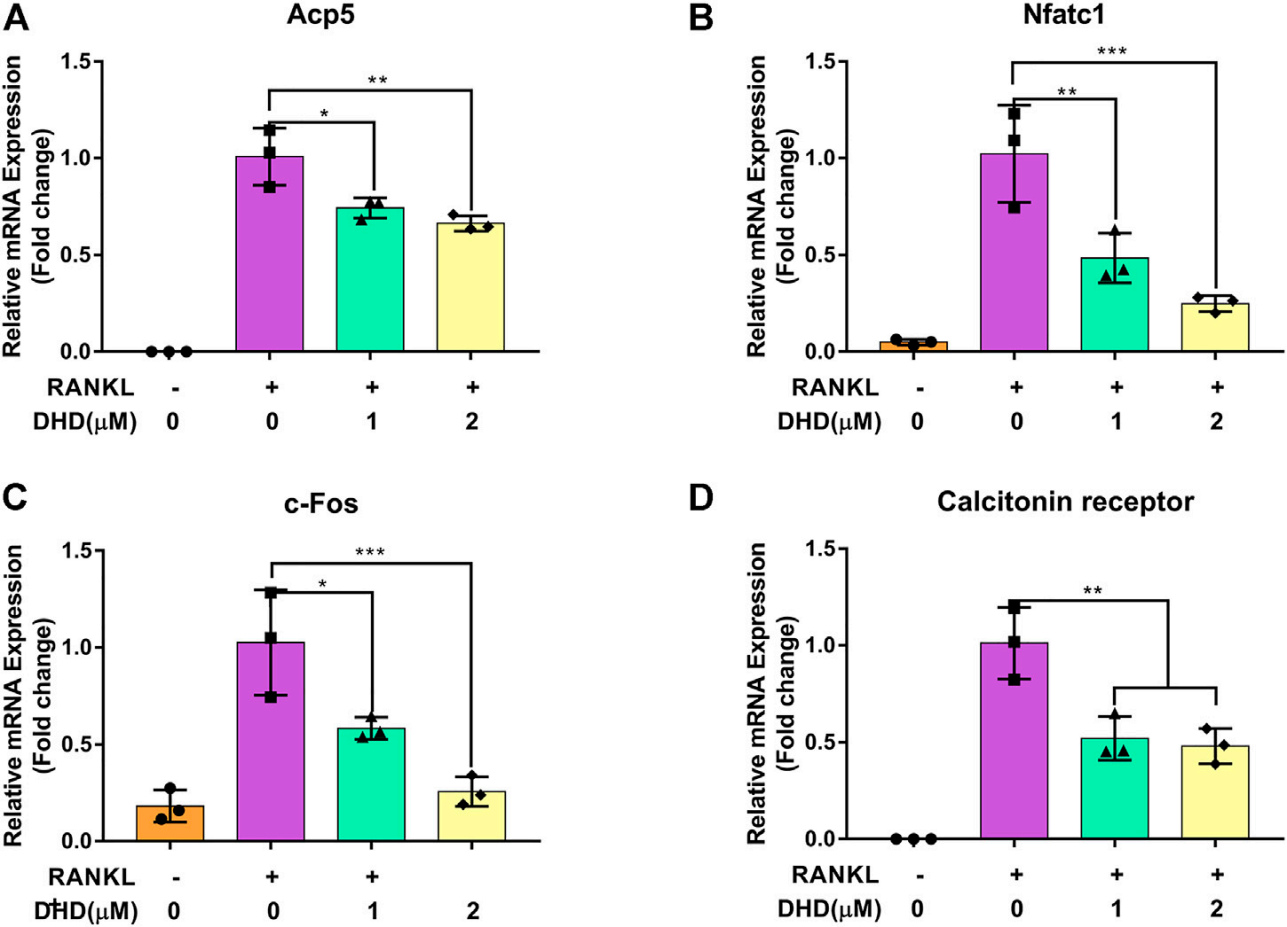
DHD blocks osteoclasts-specific gene expression. **(A)** Acp5, **(B)** NFATc1, **(C)** c-Fos, and **(D)**Ctr.

Apart from the test of osteoclasts formation, we further appraised whether DHD reduces osteoclasts bone resorptive function through the hydroxyapatite resorption assay. Mature osteoclasts were transferred to the hydroxyapatite-coated plate and exposed to DHD at a dose of 1 and 2 μM for 48 h. We discovered that the area of hydroxyapatite resorption and the number of osteoclasts per well were dramatically decreased in comparison to the RANKL alone group (Figures 4A–C). These outcomes indicated that DHD plays an important role in attenuating the resorption activity of osteoclasts. Additionally, the osteoclasts-specific genes, including *Acp5*, *Nfatc1*, *c-Fos*, and calcitonin receptor (*Ctr*), were also estimated *via* RT-qPCR. As depicted in Figures 5A–D, the mRNA expression levels of the above genes were significantly downregulated after 1 and 2 μM DHD treatment. Taken together, these data indicated that DHD could also attenuate the hydroxyapatite resorption and the expression of osteoclasts-specific genes *in vitro* asides from osteoclasts formation in a dose-dependent manner.

### DHD Eliminates RANKL-Mediated NFATc1-Related Signaling Pathway

**FIGURE 6:**
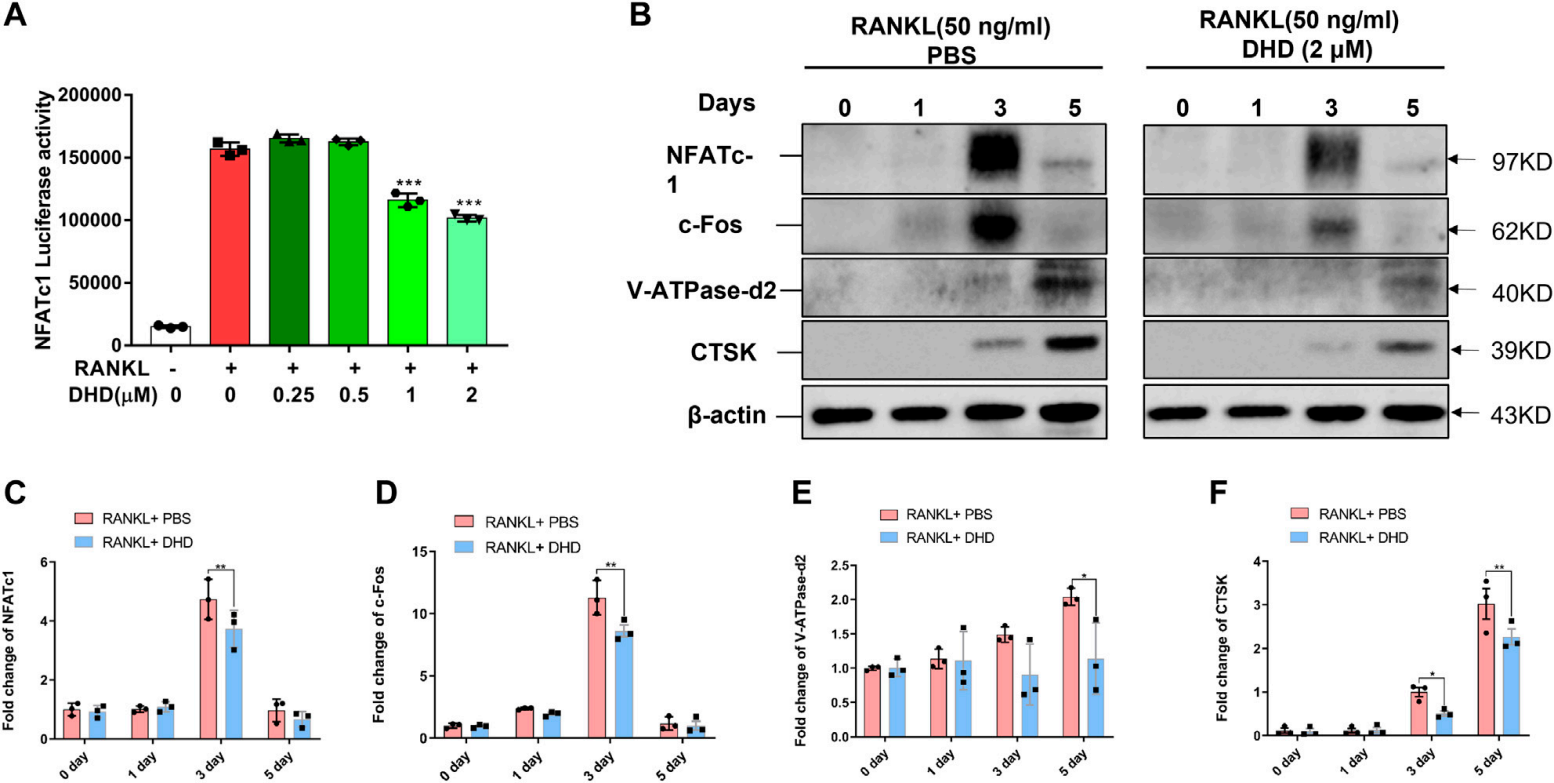
DHD represses NFATc1 activity and downstream protein expression. **(A)** Bar graph depicting luciferase activity of RAW264.7 cells stably transfected with the NFATc1 luciferase reporter construct. **(B)** Representative Western blotting images of NFATc1, c-Fos, V-ATPase-d2, and CTSK on days 0, 1, 3, and 5 after stimulation by RANKL (50 ng/ml) with or without DHD (2 μM). **(C–F)** The intensity of the band for all the proteins mentioned above was calculated as a ratio to *β*-actin.

To clearly understand the intracellular molecular mechanism of DHD suppressing osteoclasts formation and bone resorption, we conducted an in-depth study on the DHD effect on signaling pathways mediated by RANKL. RANKL activates NFATc1, which is pivotal for osteoclasts differentiation. Therefore, we wanted to determine if DHD inhibitory effect on NFATc1 activity. First, RANKL, in combination with various DHD concentrations, promoted RAW264.7 cells transduced with the NFATc1 luciferase reporter construct. As presented in Figure 6A, DHD treatment at concentrations of 1 and 2 μM had significantly reduced NFATc1 luciferase activity. Western blot analysis level was utilized to determine the protein for confirmation of the luciferase reporter assay results. The NFATc1 protein expression had been remarkably upregulated induced by RANKL stimulation, while DHD significantly inhibited its expression (Figures 6B,C). Concurrently, DHD was also found to restrict the c-Fos protein expression, an NFATc1 vital regulator, on day 3 as well (Figures 6B,D). Additionally, the NFATc1 expression associated with downstream protein, including V-ATPase-d2 and cathepsin K (CTSK), was also shown as a downswing after the DHD treatment (Figure 6B,E,F). In summary, these findings revealed that DHD possesses a significant inhibitory effect on NFATc1 and its related signaling pathway, both of which are indispensable for the functional differentiation of osteoclasts.

### DHD Suppresses RANKL-Induced Activation of MAPK Pathway

**FIGURE 7:**
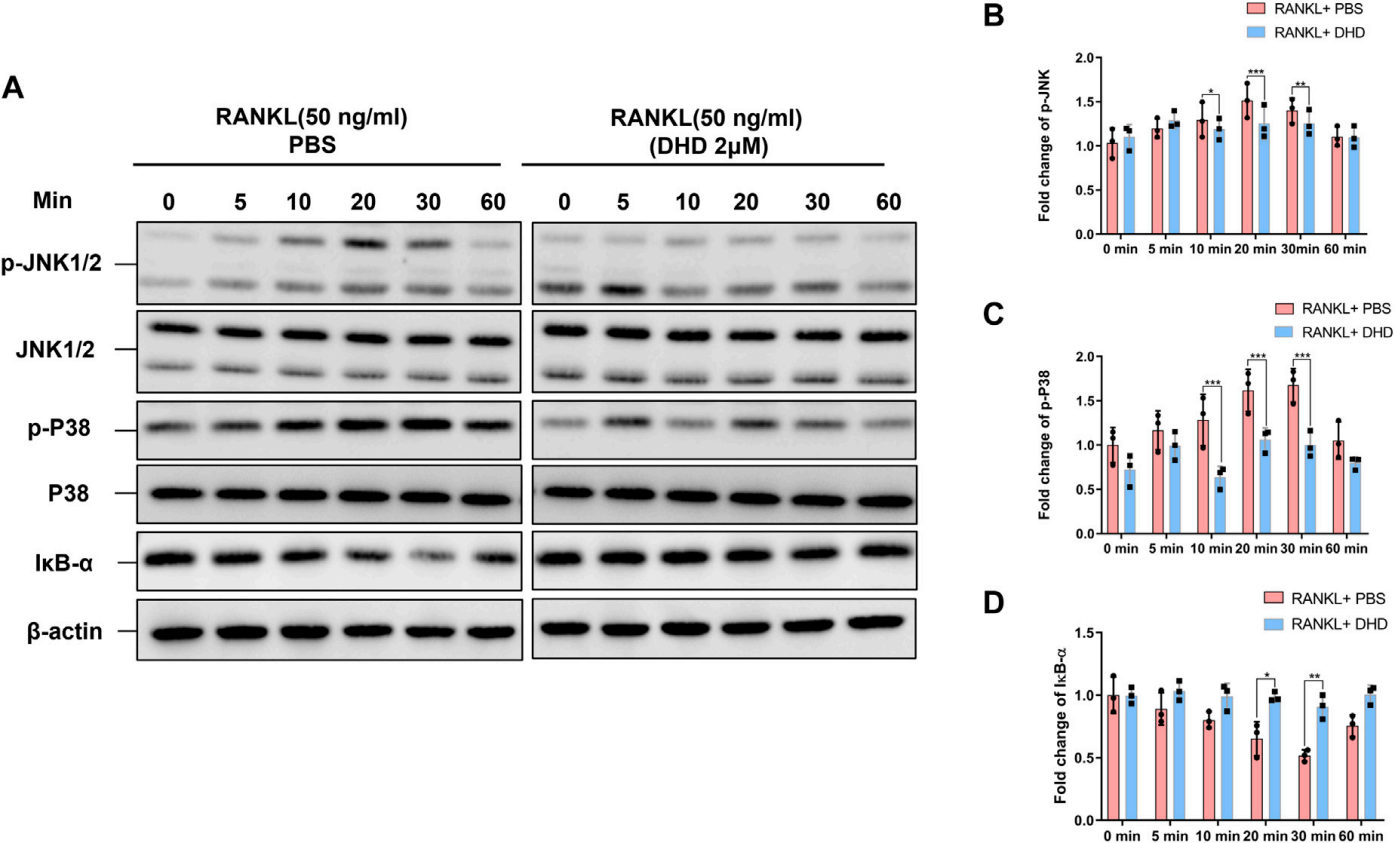
DHD inhibits the RANKL-induced MAPK signaling pathways. **(A)** Representative Western blotting images of the effects of DHD on phosphorylation JNK1/2 and P38 and IκB-α. DHD was used to pretreat BMMs for 1 h followed by 0, 5, 10, 20, 30, and 60 min of RANKL (50 ng/ml) stimulation. **(B–D)** Quantitative analysis of the fold change in p-JNK, p-P38, and IκB-α expression after DHD (2 μM) treatment.

As noted, the RANKL-induced MAPK pathway plays a crucial role in the regulation and activation of NFATc1. In order to explore how DHD affects the MAPK pathway during osteoclastogenesis, WB was utilized to determine the p-JNK1/2 and p-P38 protein expression at 0, 5, 10, 20, 30, and 60 min following 50 ng/ml RANKL induction in the presence and absence of DHD (2 μM). At 20 min, DHD exhibited the strongest inhibitory effect on the expression of p-JNK1/2 (Figures 7A,B). Besides, the phosphorylation of P38 was conspicuously conquered at 10, 20, and 30 min with DHD treatment. However, no pronounced differences in p-ERK1/2 expression were observed among the groups (Supplementary Figure S1). The nuclear factor of the kappa light polypeptide gene enhancer in B-cell inhibitor, alpha (IκB-α) protein, is one member of a family of cellular proteins, inhibiting the activity of NF-κB transcription factor. As illustrated in Figure 7A,D, the degradation of IκB-α was prohibited at 20 and 30 min in comparison to the RANKL + PBS group with DHD treatment which enlightened the potential role of DHD in the NF-κB pathway. In conclusion, these findings revealed that DHD serves as an inhibitor to suppress the RANKL-induced MAPK (including p-JNK1/2 and p-P38) pathways.

### DHD Inhibits RANKL-Induced Calcium Oscillations

**FIGURE 8:**
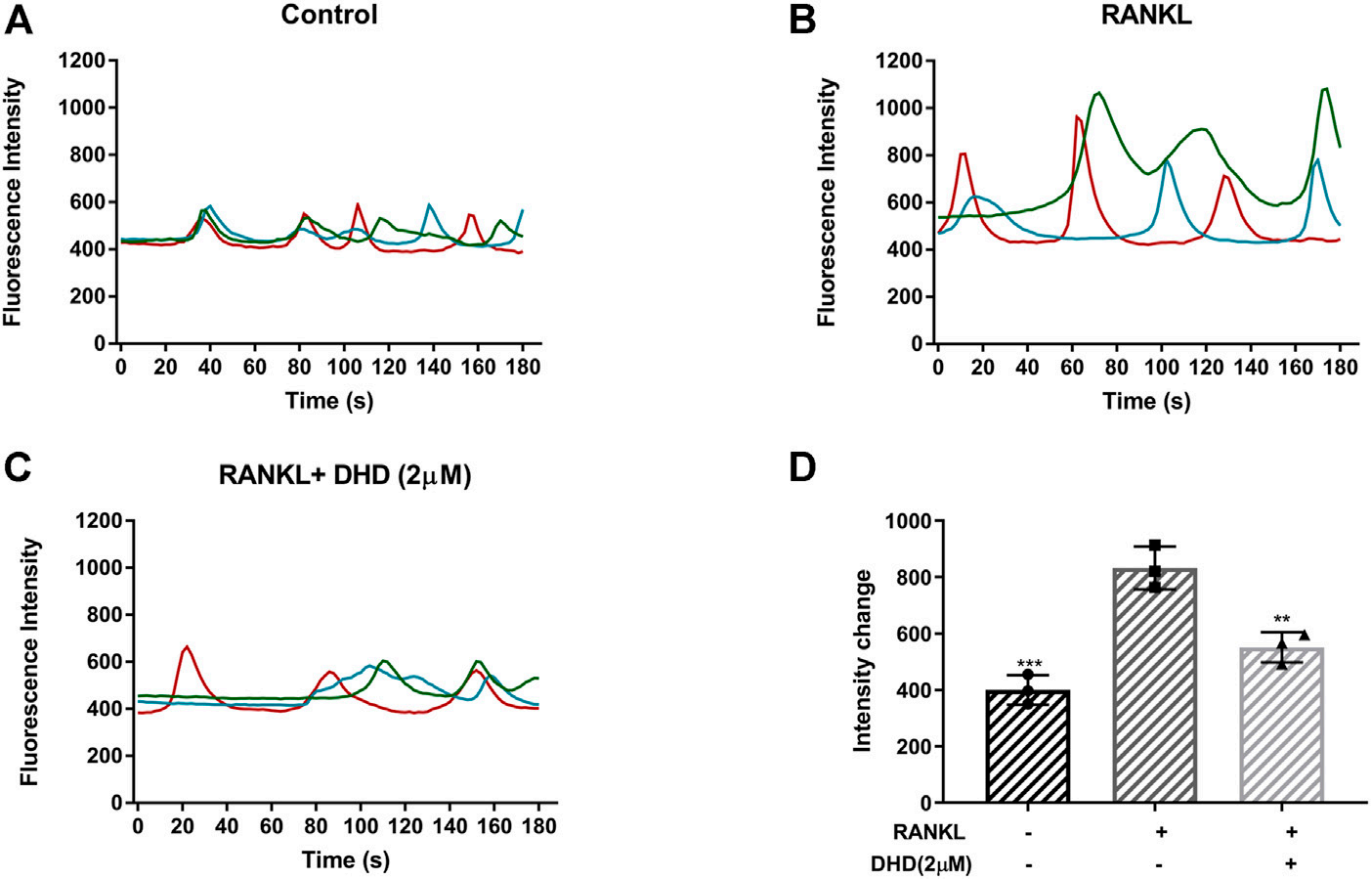
DHD inhibits RANKL-induced calcium oscillations. Representative images of fluorescence intensity of intracellular Ca^2+^ oscillation in **(A)** RANKL group, **(B)** control group, and **(C)** RANKL + DHD (2 μM) group. Different colored lines in each image indicate the results of three independent experiments. **(D)** The fluorescence intensity of calcium oscillation in three groups was quantitatively analyzed (*n* = 3). Fluorescence intensity change was measured by subtracting the minimum peak intensity from the maximum peak intensity.

Ca^2+^ oscillations, which are initiated by RANKL in the Ca^2+^ signal transduction pathways, also activate NFATc1. As a consequence, the role of DHD in Ca^2+^ oscillations was reckoned. In accordance with our preliminary expectations, RANKL-induced Ca^2+^ oscillations were markedly reduced, by nearly 40%, with DHD intervention (Figures 8A–D). Collectively, these findings suggested that DHD attenuates the amplitude of RANKL-induced calcium oscillations.

### DHD Enhances the Expression of ROS Scavenging Enzymes

**FIGURE 9:**
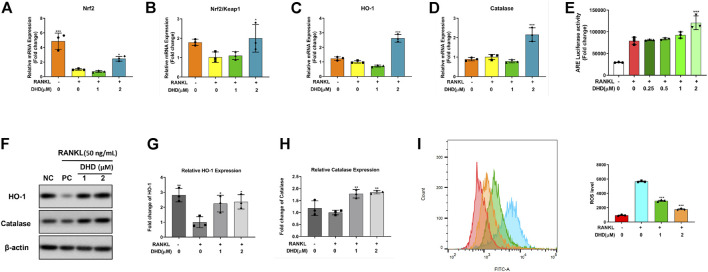
DHD increases the expression of ROS scavenging genes and proteins and ARE activity. **(A–D)** Relative mRNA expression of Nrf2, Nrf2/Keap1, Ho-1, and CAT. **(E)** DHD slightly improves the ARE luciferase activity of RAW 264.7 cells stably transfected with the ARE luciferase construct. **(F)** Representative Western blotting images of HO-1 and catalase. **(G,H)** The ratios of band intensity of the above proteins, which were relative to β-actin expression, were quantitatively analyzed. **(I)** BMMs were treated with 50 ng/ml RANKL and DHD at different concentrations (0, 1, and 2 μM), and flow cytometric analysis was conducted to quantify ROS production (mean fluorescence intensity).

The RANKL-mediated reactive oxygen species (ROS) pathway is closely linked with OCs differentiation, which was deemed a potential therapeutic target for osteoporosis (Kim et al., 2010). The antioxidant defense system, composed of antioxidant enzymes and non-enzymatic oxidants, is a critical cellular defensive mechanism against ROS (Li et al., 2017). Antioxidant enzymes such as Ho-1 and catalase (CAT) represent the primary enzymatic antioxidant defense against oxidative stress by directly neutralizing ROS. The major inducer of those cell-protective enzymes is the Nrf2-Keap1 protein axis. To further determine whether DHD could upregulate the expression of ROS-scavenging enzymes, RT-qPCR and WB were performed. We found that DHD increases the gene level of Nrf2 and the rate of Nrf2/Keap1 (Figures 9A,B). Consistently, the expression of these cell-protective enzymes was elevated following DHD treatment at the concentrations of 1 and 2 μM (Figures 9 C,D,F–H). Moreover, Nrf2 could translocate to the nucleus, where it interacts with ARE for ARE-mediated antioxidant genes stimulation (Kim et al., 2018). Hence, we transfected ARE luciferase plasmids into RAW264.7 cells and stimulated them through RANKL (50 ng/ml) with DHD at the indicated concentrations. As uncovered in Figure 9E, the activity of ARE luciferase was dramatically upregulated at a concentration of 2 μM DHD. Simultaneously, the ROS level was also notably diminished by DHD (Figure 9I). Taken together, these results exhibited that DHD enhances the ROS scavenging enzyme expression through the Nrf2/Keap1/ARE signaling pathway and plays a critical role in the inhibition of osteoclasts formation mediated by RANKL.

### DHD Prevents Bone Loss *In Vivo*

**FIGURE 10:**
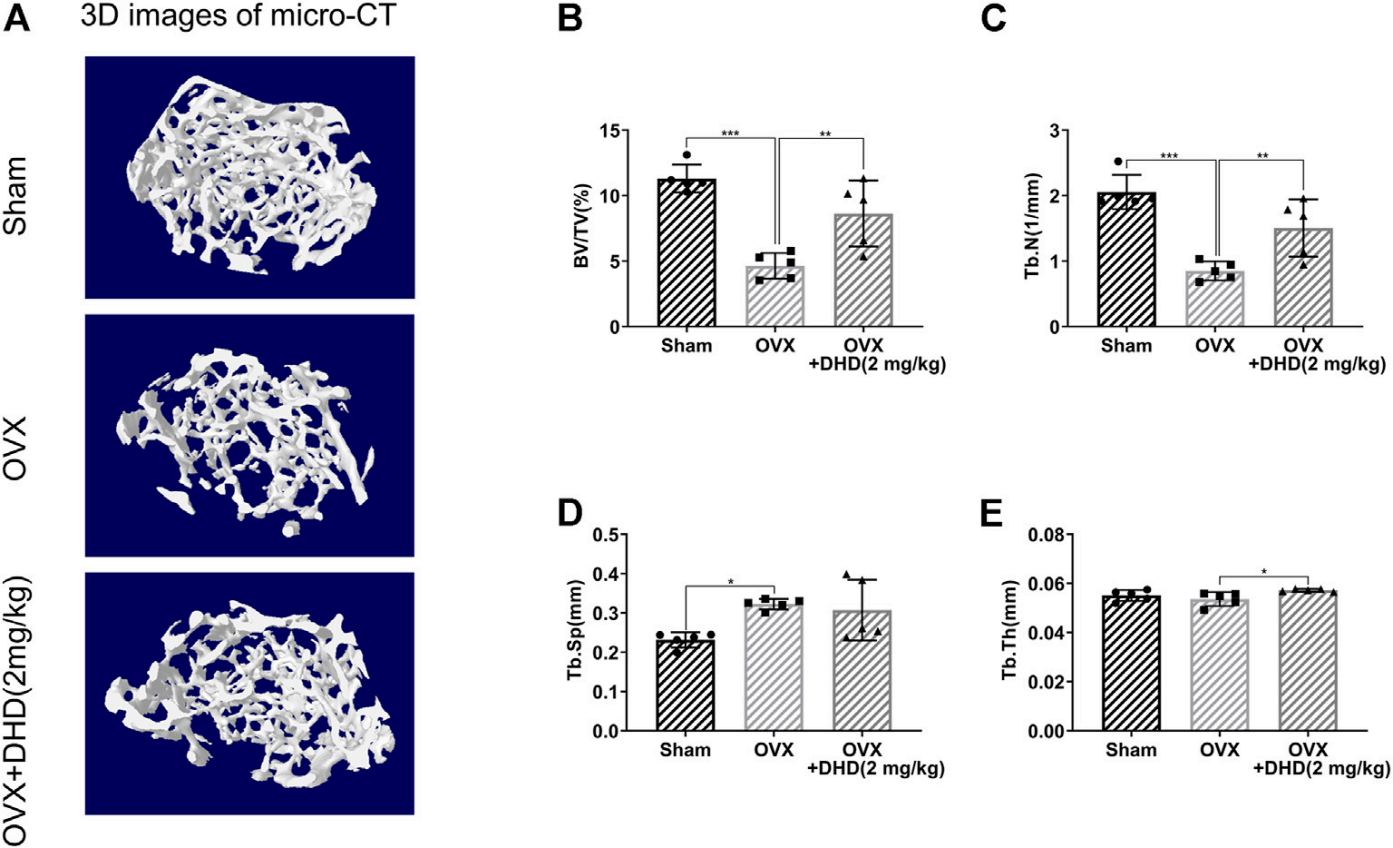
DHD ameliorates OVX-induced systemic bone loss *in vivo*. **(A)** Representative 3D reconstruction micro-CT images of femur trabecular bone in the Sham, OVX, and OVX + DHD (2 mg/kg) groups. **(B–E)** BV/TV, Tb.N, Tb.Sp, and Tb.Th were quantitatively analyzed based on micro-CT results.

Micro-CT was utilized for the femur analysis, and then several parameters affecting bone mass were qualitatively and quantitatively analyzed. Reconstructed 3D images of the femurs revealed a significant bone loss in the OVX mice, while the bone loss was alleviated in the OVX + DHD group (Figure 10A). Meanwhile, BV/TV and Tb.N were distinguishably strengthened, whereas there was no statistical difference in Tb.Sp in the OVX + DHD group compared to the OVX group (Figures 10B–E). However, Tb.Th and cortical bone parameters illustrated no statistically significant variation between the OVX and OVX + DHD groups (Supplementary Figures S2B–E). Altogether, these results manifested that DHD has a protective effect on estrogen deficiency-induced systemic bone loss.

## Discussion

Bone remodeling is an ever-occurring event to maintain mineralized balance and structural integrity (Wu et al., 2019). Harmony coordination of osteoblasts and osteoclasts activities during bone remodeling plays an essential role in maintaining the dynamic balance of bone (Jadhav et al., 2016). Once this equilibrium is broken, osteoporosis can be caused by excessive bone resorption activity mediated by overactivated osteoclasts (Chen et al., 2017). In view of the reality that current managements for osteolytic diseases are concomitant with several adverse effects, including osteonecrosis and hormonal disorders, which curb their clinical application, the exploration of novel medications targeting overactivated osteoclasts may broaden current clinical osteoporosis treatment protocols. The concerns about compounds from Chinese herbal medicine raised in recent years ascribe to their wide spectrum of biological activity and rare undesirable effects. DHD belongs to the diterpenoid compounds, which have higher biological activity. However, there are extremely few studies about the effect of DHD on bone metabolism, such as osteoporosis. Under this context and the guidance of the pharmacological consensus, we first testified that DHD could dramatically inhibit the biological function of the osteoclasts, including cell differentiation, the F-actin belt formation, and bone resorption activity without any obvious cytotoxicity. Mechanistically, DHD exerted those functions by abrogating RANKL-induced MAPK, calcium, and NFATc1 signaling pathway and promoting the expression of ROS scavenging enzymes. Furthermore, we demonstrate a protective effect of DHD against bone loss *in vivo* by an OVX-induced osteoporosis mouse model. Our findings demonstrated that DHD could serve as a promising natural pharmaceutical agent for treating osteoporosis.

Osteoclasts are multinucleated cells originating from hematopoietic progenitors in the bone marrow. Various cytokines have been displayed to induce osteoclasts formation, which includes M-CSF, RANKL, and interleukin-1 (IL-1). Among these, RANKL, interacting with its receptor RANK, has been considered the principal osteoclasts differentiation factor. RANKL/RANK complex activates NFATc1, a crucial transcription factor in the osteoclastogenesis process, by recruiting tumor necrosis factor receptor-associated factor 6 (TRAF6) to not only govern the osteoclastogenesis but also regulate the resorption activity of osteoclasts (Kobayashi et al., 2001). As a pivotal signaling pathway of osteoclastic differentiation and function, RANKL-mediated NFATc1 activation has emerged as a novel target for the treatment of osteoporosis (Vihma et al., 2008; Zhao et al., 2010). Our results manifested that NFATc1 activity was remarkably blocked by DHD in RANKL-induced BMMs. In addition, the NFATc1 promoter region could also be mediated by other transcription factors, such as c-Fos and NF-κB.

C-Fos, an AP-1 component, is an essential factor in activating several target genes (Acp5, V-ATPase-d2, Ctsk, and Ctr) involved in OCs differentiation and function by cooperating with NFATc1 (Shen et al., 2007; Balkan et al., 2009). In our present study, DHD suppressed the protein expression of c-Fos and the expression of their downstream genes. MAPK is closely related to osteoclastogenesis induced by RANKL and regulates the downstream nuclear factors c-Fos and NFATc1 (Wong et al., 1998; Matsuo et al., 2004; Kim and Kim, 2014). The MAPK family is made up of three major parallel pathways: the ERK, JNK, and P38 pathways (Wei et al., 2013; Park et al., 2017). JNK and P38 MAPK signaling pathways are predominantly responsible for OCs formation, while ERK is crucial for OCs survival (Lee et al., 2001). The JNK, P38, and ERK are all phosphorylated in response to RANKL stimulation. Our study revealed that the protein expression of phosphorylation of JNK and P38 was suppressed by DHD, whereas the expression of ERK was not impaired. Those findings suggested that DHD maybe only suppress the osteoclastogenesis other than OCs survival. NF-κB activation is one of the downstream events after the stimulation of RANKL (Ono and Nakashima, 2018). Mounting evidence suggested that the activity of the NF-κB transcription factor was inhibited by IκB-α (Karin and Ben-Neriah, 2000). The degradation of IκB-α was inhibited by DHD, which indicates the NF-κB pathway maybe also participated in the OCs differentiation in our study. However, the specific effect of DHD on the NF-κB complex will be further investigated. What is more, accumulating data revealed that NFATc1 is a calcium-dependent neurocalcin, which was activated and automatically amplified by calcium oscillations promoted by RANKL. Apparently, the calcium signaling pathway is engaged in NFATc1 regulation (Zhou et al., 2011; Kajiya, 2012; Wang et al., 2019). Our results illustrated that DHD plays its inhibitory effect on osteoclastogenesis by diminishing the mean amplitude of calcium oscillation.

Recently, ongoing evidence suggested that ROS and calcium signaling pathway are closely linked to RANKL-dependent osteoclastogenesis (Callaway and Jiang, 2015; Zhou et al., 2017; Agidigbi and Kim, 2019) (Ha et al., 2004; Kim et al., 2010; Zhang et al., 2016). More specifically, various ROS negative regulators, including Nrf2, Ho-1, and catalase, have also been shown to be involved in this process (Ishii et al., 2000; Mlakar et al., 2012; Sun et al., 2015; Anselmino et al., 2020). Consequently, we detected whether DHD exhibits an antioxidant effect during osteoclasts formation. As expected, the expression levels of Nrf2, Ho-1, and catalase were remarkably increased in a dose-dependent manner to DHD. Besides, the Nrf2/Keap1 ratio reflects the ability of ROS scavengers (Bellezza et al., 2018). In consistence with the above results, DHD could prominently prompt the ratio of Nrf2/Keap1. Furthermore, the ability of Nrf2 to degrade ROS could be achieved through binding to ARE, which is in the nucleus. Interestingly, we found that transcriptional activity of ARE was strikingly elevated by DHD.

Finally, we conducted an *in vivo* study through the OVX mouse osteoporosis model for further confirmation of the potential therapeutic effect of DHD in reducing systemic bone loss. Our data revealed that BV/TV, Tb.Th, and Tb.N were elevated in the DHD treatment group. In contrast, we did not find significant cortical bone loss in OVX mice, which is consistent with previous studies (Nakamura et al., 2007). As expected, no significant effect of DHD on cortical bone analytical parameters was observed in our study. Although our results confirmed that DHD might be a promising agent in treating osteoporosis, DHD is likely to have potential toxicity. In order to address this issue, we are planning to introduce the liposome-assisted drug delivery system to minimize the possible toxicity of DHD for future clinical translation. As a novel type of drug carrier, liposome could efficiently accomplish drug sustained-release to minimize the DHD potential toxicity (Vickerman et al., 2021). In future studies, the experiment will be further explored, and our research group will also focus on the role of liposomes.

## Conclusion

**FIGURE 11:**
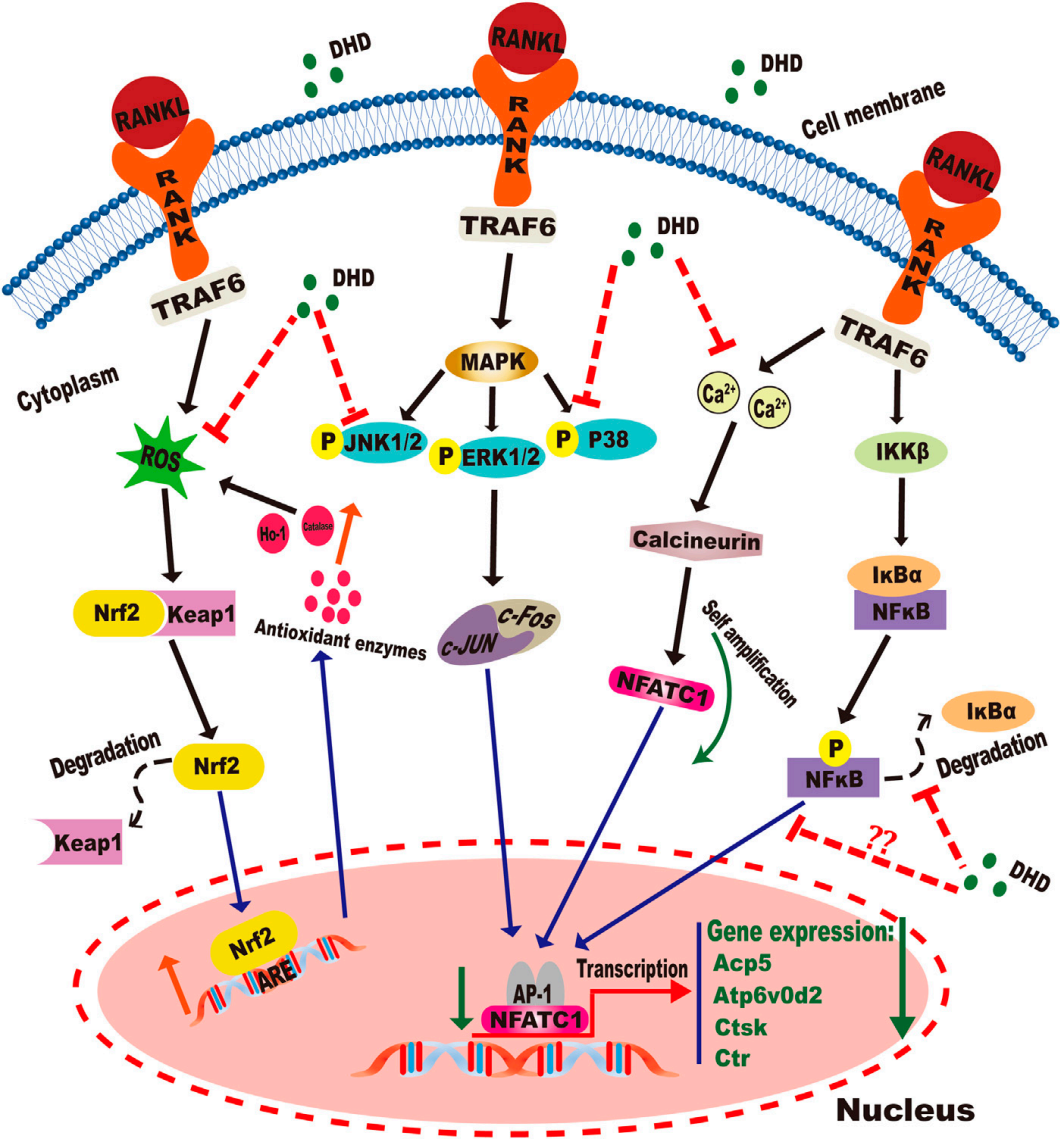
Graphical abstract for illustrating the role of DHD in repressing RANKL-mediated osteoclastogenesis through inhibition of MAPK, calcium, and Nrf2/Keap1/ARE signaling pathways.

Our data confirmed that DHD could inhibit osteoclastogenesis by abrogating RANKL-induced MAPK, calcium, and NFATc1 signaling pathways and promoting the expression of ROS scavenging enzymes, thereby preventing OVX-induced bone loss (Figure 11). Therefore, this study demonstrates that DHD may act as a novel therapeutic agent to manage osteoporosis in clinical practice.

## Data Availability

The raw data supporting the conclusion of this article will be made available by the authors without undue reservation.

## Supplementary Material

Supplementary Figure S1 Representative Western blotting images of the effects of DHD on ERK1/2 and p-ERK1/2. DHD was used to pretreat BMMs for 1 h followed by 0, 5, 10, 20, 30, and 60 min of RANKL (50 ng/ml) stimulation.

Supplementary Figure S2 DHD had no significant effect on the femoral cortical bone in vivo. **(A)** Representative 3D reconstruction micro-CT images of femur cortical bone in the three groups. **(B–E)** Quantitative measurements of femur cortical bone-related parameters, such as Ct.Ar, Tt.Ar, Ct.Ar/Tt.Ar, and Ct.Th.

## Abbreviations

ARE, antioxidant response element; BMMs, bone marrow monocytes; BSA, bovine serum albumin; BV/TV, bone volume/tissue volume; Ct.Ar, cortical bone area; Ct.Ar/Tt.Ar, cortical area fraction; Ct.Th, cortical thickness; Ctr, calcitonin receptor; CTSK, cathepsin K; DHD, 12-deoxyphorbol-13-hexadecanoate; F-actin, fibrous actin; FBS, fetal bovine serum; MAPK, mitogen-activated protein kinase; M-CSF, macrophage colony-stimulating factor; NFATc1, nuclear factor of activated T cells 1; OVX, ovariectomy; PBS, phosphate-buffered saline; RANKL, receptor activator of the nuclear factor-κB ligand; ROS, reactive oxygen species; Tb.N, trabecular number; Tb.Sp, trabecular spacing; Tb.th, trabecular thickness; TCM, Traditional Chinese Medicine; TRAF6, tumor necrosis factor receptor-associated factor 6; Tt.Ar, total tissue area; VOI, volume of interested.
