# Supplementary figures and images for: 12-Deoxyphorbol-13-Hexadecanoate Abrogates OVX-Induced Bone Loss in Mice and Osteoclastogenesis via Inhibiting ROS Level and Regulating RANKL-Mediated NFATc1 Activation
Source: Front Pharmacol. 2022 Jun 3;13:899776. doi: 10.3389/fphar.2022.899776 (PMC9204068; doi:10.3389/fphar.2022.899776)

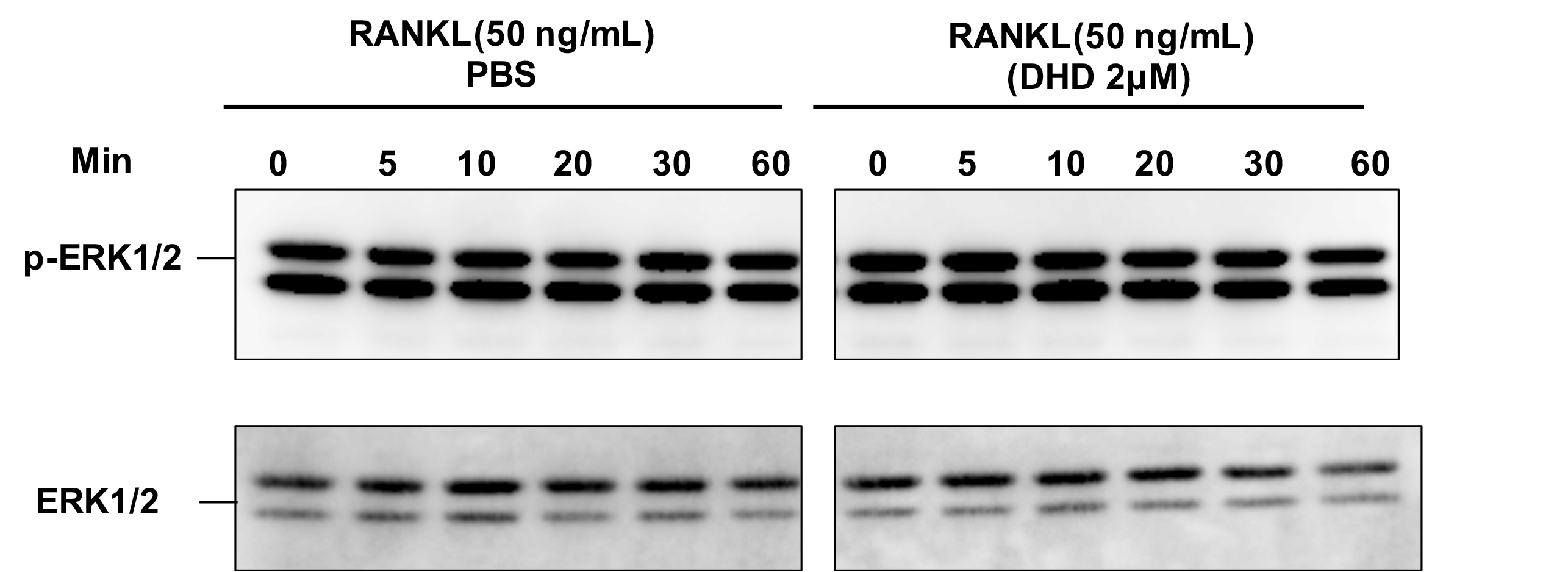

Supplement: Supplementary file 2 [file Image2.TIF]

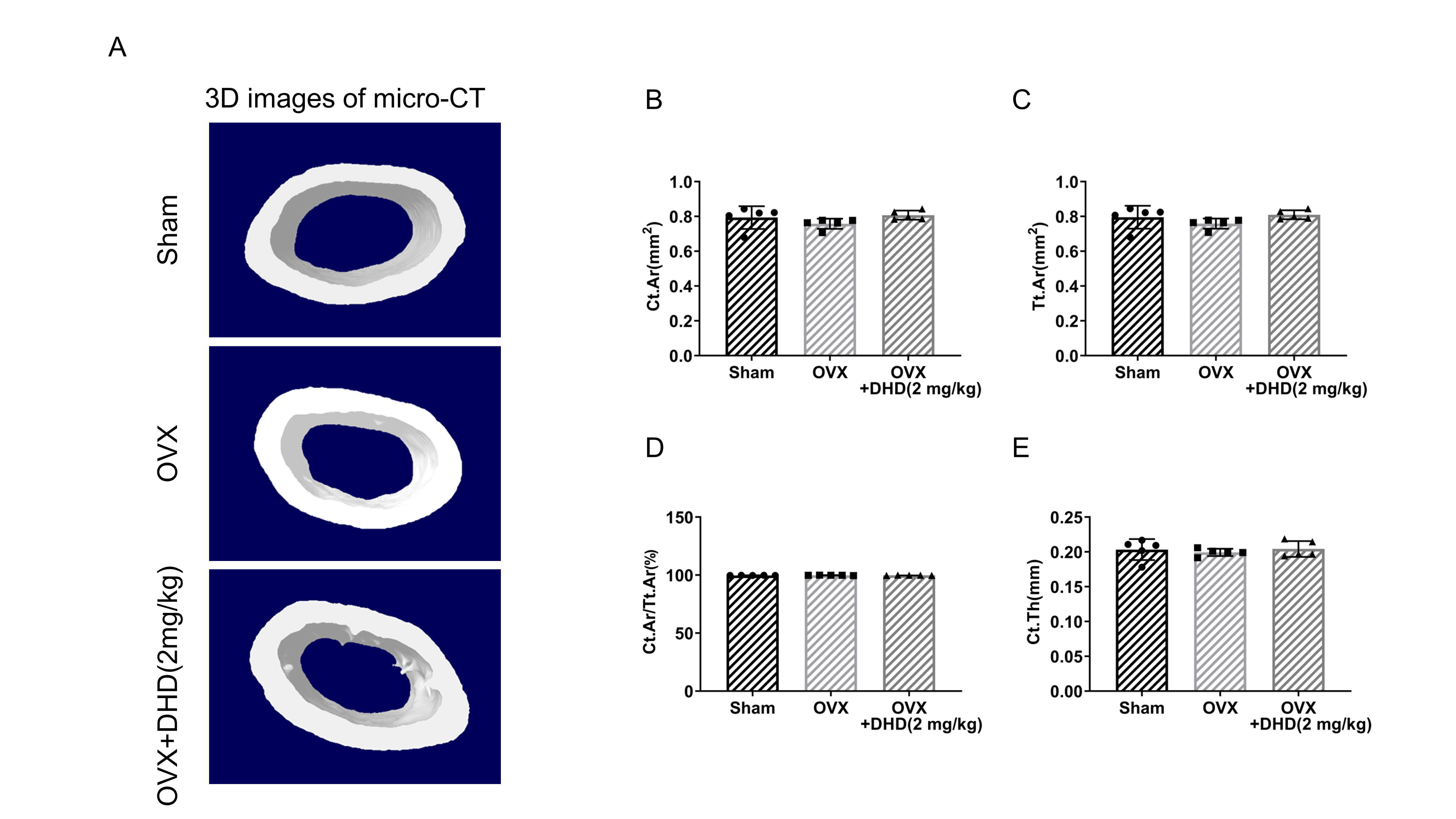

Supplement: Supplementary file 3 [file Image1.TIF]
